# Supplementary material for: Structural recognition and stabilization of tyrosine hydroxylase by the J-domain protein DNAJC12
Source: Nat Commun. 2025 Mar 20;16:2755. doi: 10.1038/s41467-025-57733-6 (PMC11926245; doi:10.1038/s41467-025-57733-6)

Supplementary Information

Structural recognition and stabilization of tyrosine hydroxylase by the J-domain protein DNAJC12

Mary Dayne S. Tai[1,2], Lissette Ochoa[3], Marte I. Flydal[1,4], Lorea Velasco-Carneros[5], Jimena Muntaner[3], César Santiago[3], Gloria Gamiz-Arco[1,2], Fernando Moro[5], Kunwar Jung-KC[1,2,6], David Gil-Cantero[3], Miguel Marcilla[3], Juha P. Kallio[1], Arturo Muga[5], José María Valpuesta[3,7,*], Jorge Cuéllar[3,*], Aurora Martinez[1,2,6,*]

[1]Department of Biomedicine, University of Bergen, Bergen, Norway.

[2]Neuro-SysMed Center, Department of Neurology, Haukeland University Hospital, Bergen, Norway.

[3]Centro Nacional de Biotecnología (CNB-CSIC), Madrid, Spain.

[4]Department of Medical Genetics, Haukeland University Hospital, Bergen, Norway.

[5]Instituto Biofisika (UPV/EHU, CSIC), Universidad del País Vasco, (UPV/EHU), and Departamento de Bioquímica y Biología Molecular, Facultad de Ciencia y Tecnología, Universidad del País Vasco, (UPV/EHU), Barrio Sarriena, 48940 Leioa, Spain.

[6]K.G Jebsen Center for Translational Research in Parkinson's Disease, University of Bergen, Bergen, Norway.

[7]Unidad de Nanobiotecnología, CNB-CSIC-IMDEA Nanociencia Associated Unit, 28049 Madrid, Spain

*Co-corresponding authors: José María Valpuesta, jmv@cnb.csic.es; Jorge Cuéllar, jcuellar@cnb.csic.es; Aurora Martinez, aurora.martinez@uib.no.

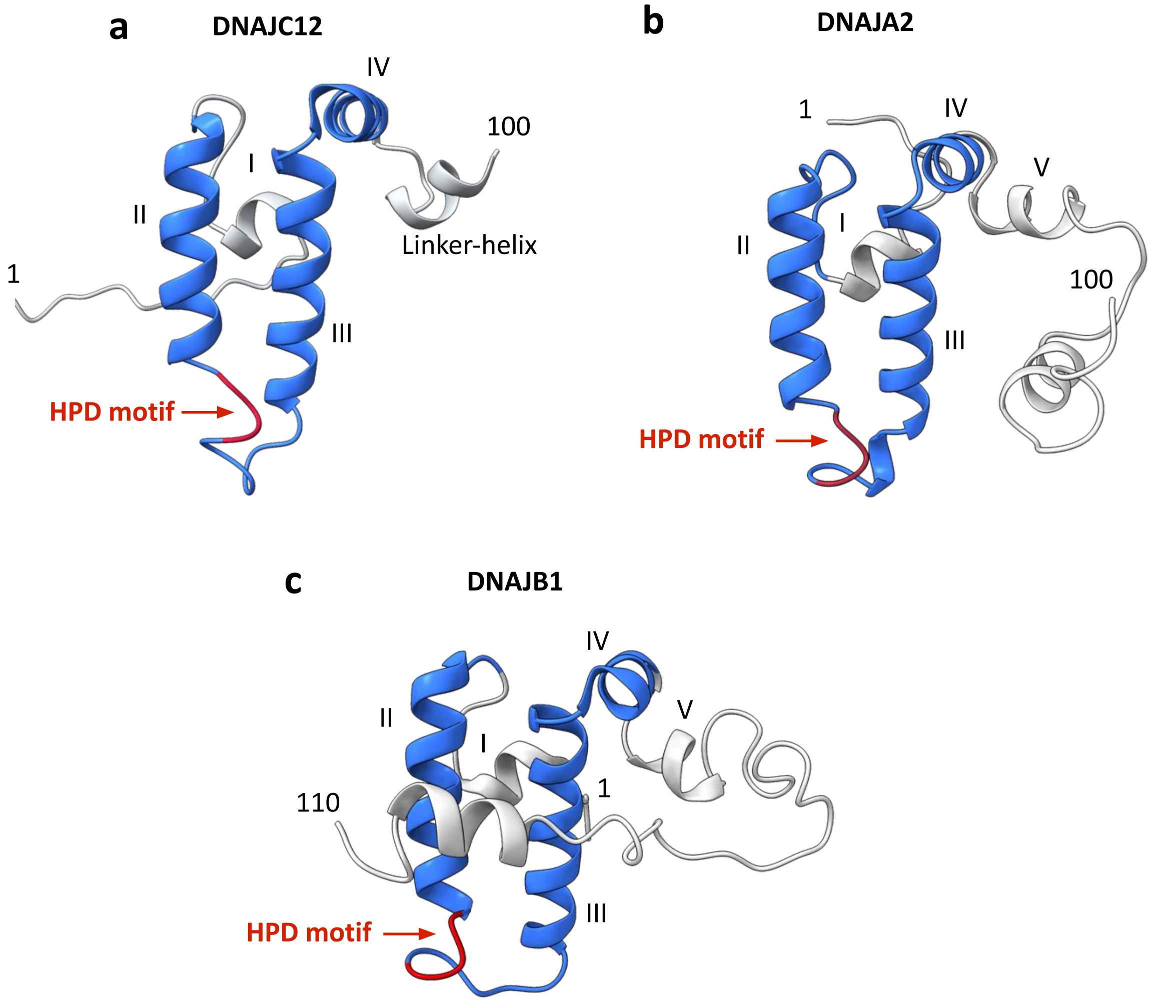

Supplementary Figure 1. The J-domain of DNAJC12 (PDB 2CTQ), DNAJA2 (PDB 7ZHS), and DNAJB1 (PDB 6Z5N).

The J-domain (JD) (first 100 resides) of **a)** DNAJC12 (NMR solved; PDB 2CTQ), **b)** DNAJA2 (AF-O60884-F1)[1] and **c)** DNAJB1 (NMR solved; PDB 6Z5N)[2]. For DNAJC12, the JD shows an unstructured 13-residue N-terminal tail, the characteristic tetra-helical structure (labelled as helices I-IV) of the JD, and an additional fifth helix (linker-helix in this work), which is incomplete in this NMR structure.

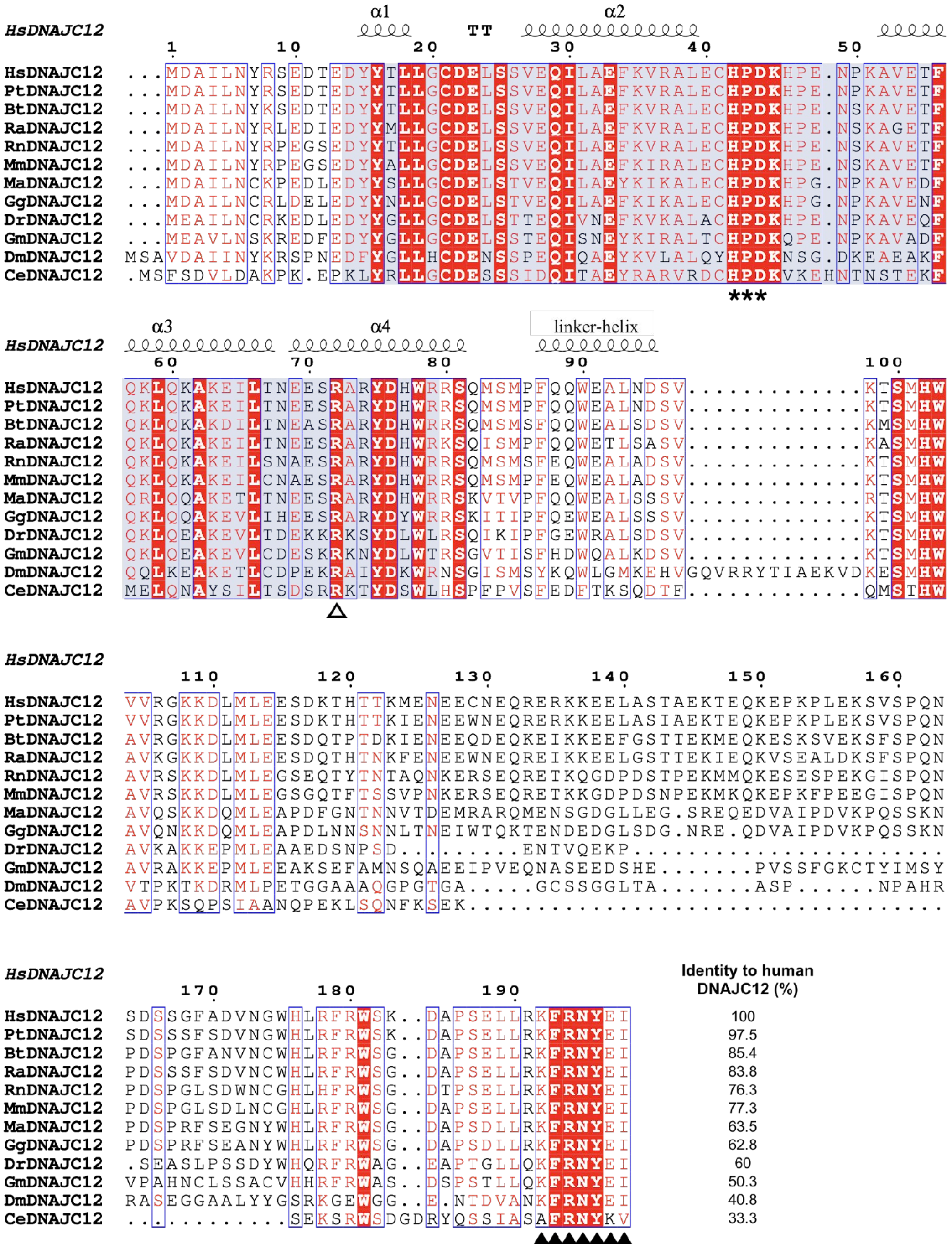

Supplementary Figure 2. Sequence alignment of DNAJC12 from different species.

Sequence alignment performed with Clustal Omega[3] and visualized with ESPript[4]. The secondary structural elements for the JD shown at the top of the alignment are extracted by ESPript from the AlphaFold structure of the full-length human DNAJC12 (AF-Q9UKB3-F1). The aligned sequences are from *Homo sapiens* (Hs; NP_068572.1), *Pan troglodytes* (Pt; XP_521687.2), *Bos taurus* (Bt; NP_776521.1), *Rousettus aegyptiacus* (Ra; XP_015985286.2), *Rattus norvegicus* (Rn; NP_001029204.1), *Mus musculus* (Mm; NM_013888.3), *Motacilla alba* (Ma; XP_037996268.1), *Gallus gallus* (Gg; NP_001186459.1), *Danio rerio* (Dr; NP_001314717.1), *Gadus morhua* (Gm; XP_030235349.1), *Drosophila melanogaster* (Dm; NP_651807.1), *Caenorhabditis elegans* (Ce; NP_001023271.1). Residues strictly conserved across all sequences are marked with red background, and residues in red letters are above the Similarity Global Score of 0.7. The C-terminal heptapeptide sequence KFRNYEI is conserved beyond vertebrates (black triangles), while FRNY is already present in *Caenorhabditis elegans*. The JD is highlighted in gray, while the HPD motif is marked with asterisks. Arg72, which is mutated in some patients with DNAJC12 deficiency, is conserved in all DNAJC12 homologues and is marked with a white triangle.

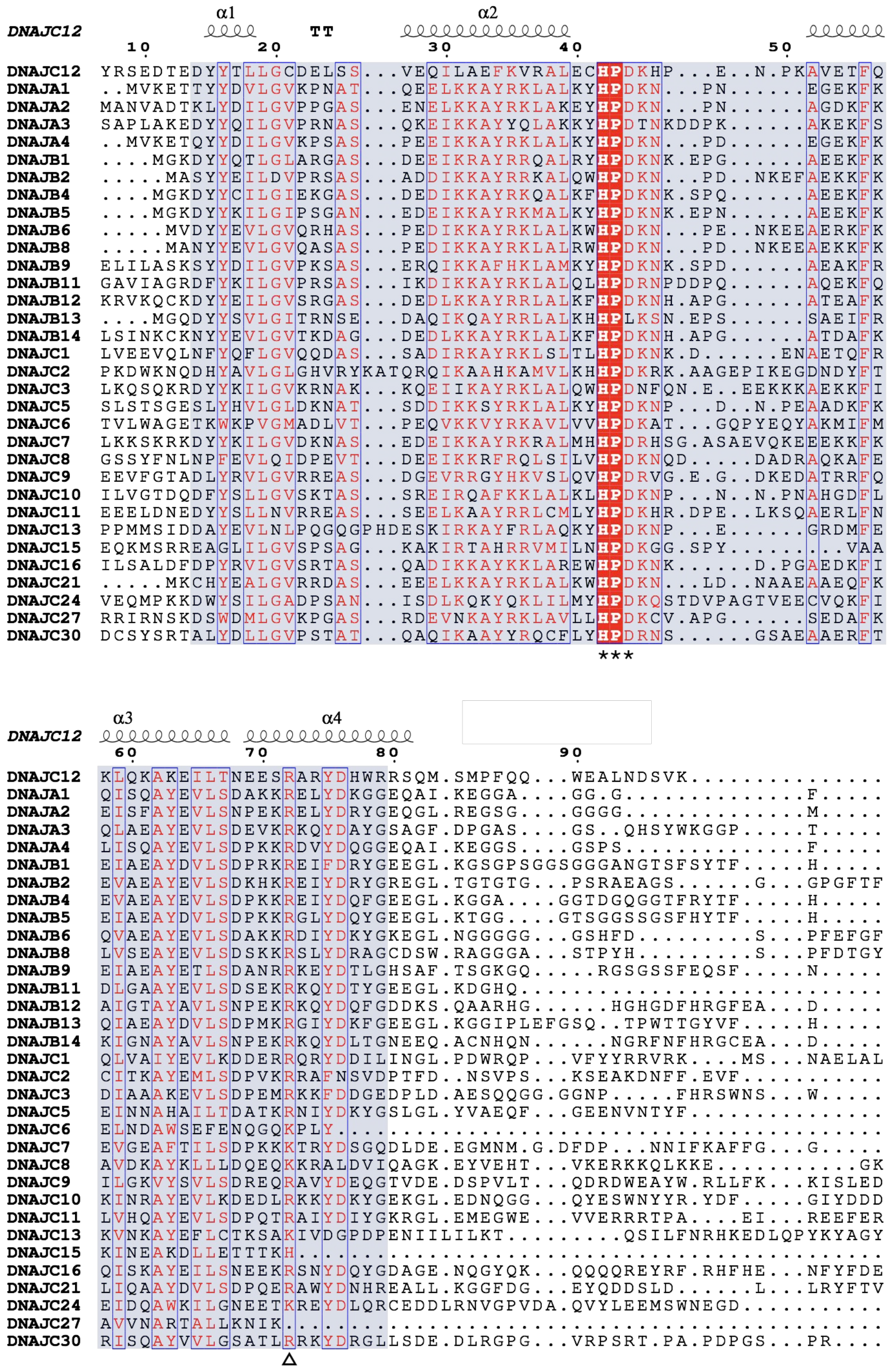

Supplementary Figure 3. Sequence alignment of the J-domain from different human JDPs.

Sequence alignment performed with Clustal Omega[3] and visualized with ESPript[4]. The secondary structural elements for the JD shown at the top of the alignment are extracted by ESPript from the AlphaFold structure of the full-length human DNAJC12 (AF-Q9UKB3-F1). The aligned sequences are DNAJC12 (NP_068572.1), DNAJA1 (NP_001530.1), DNAJA2 (NP_005871.1), DNAJA3 (NP_005138.3), DNAJA4 (NP_001123654.1), DNAJB1 (NP_006136.1), DNAJB2 (NP_006727.2), DNAJB4 (NP_001304028.1), DNAJB5 (NP_001336653.1), DNAJB6 (NP_490647.1), DNAJB8 (NP_699161.1), DNAJB9 (NP_036460.1), DNAJB11 (NP_057390.1), DNAJB12 (NP_001002762.3), DNAJB13 (NP_705842.2), DNAJB14 (NP_001026893.1), DNAJC1 (NP_071760.2), DNAJC2 (NP_055192.1), DNAJC3 (NP_006251.1), DNAJC5 (NP_079495.1), DNAJC6 (NP_055602.1), DNAJC7 (NP_003306.3), DNAJC8 (NP_055095.2), DNAJC9 (NP_056005.1), DNAJC10 (NP_061854.1), DNAJC11 (NP_060668.2), DNAJC13 (NP_056083.3), DNAJC15 (NP_037370.2), DNAJC16 (NP_056106.1), DNAJC21 (NP_001012339.2), DNAJC24 (NP_859057.4), DNAJC27 (NP_057628.1), and DNAJC30 (NP_115693.2). Residues strictly conserved across all sequences are marked with red background and red letters are above the Similarity Global Score of 0.7. The JD is highlighted in gray, while the HPD motif is marked with asterisks. R72 is also conserved in the selected human JDPs and is marked with a white triangle.

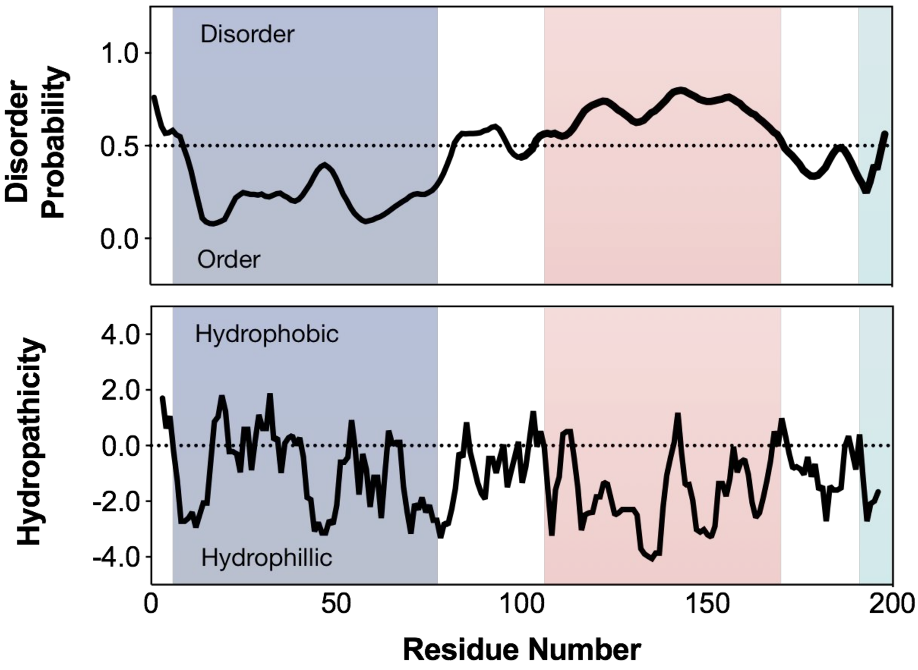

Supplementary Figure 4. Disorder and hydropathicity predictions for DNAJC12.

Upper panel, disorder prediction by IUPred2A[5]. The JD (blue) was correctly predicted to be structured, while a 67-residue stretch (residues 104-171; light red) was predicted to be disordered, whereas the C-terminal end of DNAJC12 (light green) is structured, which is in accordance with the AlphaFold model (Fig. 1b). Lower panel, hydropathicity index calculated by ProtScale with the Kyte-Doolittle scale[6]. Same color code as in upper panel. Source data are provided as a Source Data file.

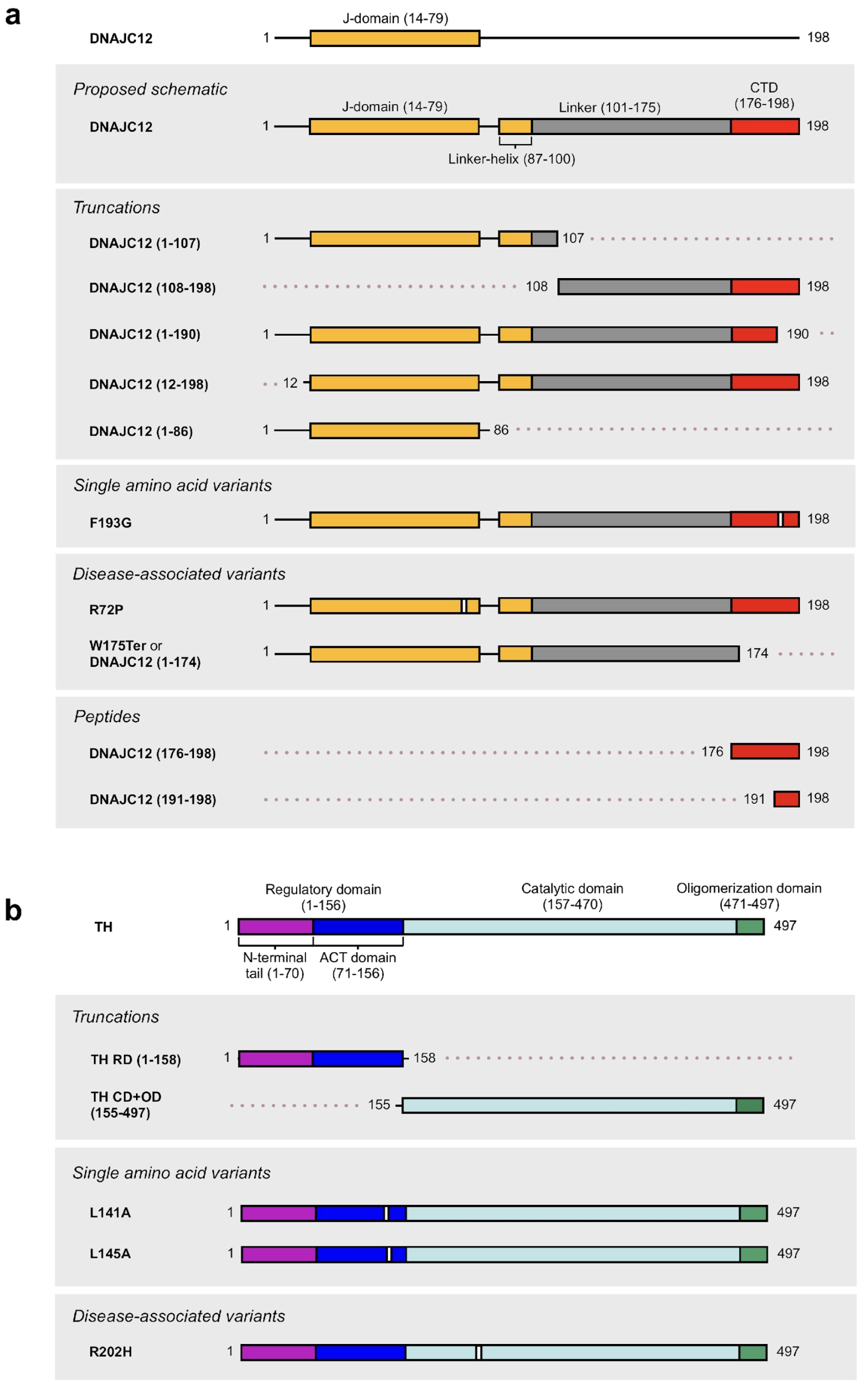

Supplementary Figure 5. Schematic representation of DNAJC12 and TH variants and peptides used in this study.

(a) Schematic representation of DNAJC12 and variants. The proposed additional functional regions in the full-length DNAJC12 sequence in addition to the JD (orange) are annotated in the representation, i.e., the linker (gray) including the linker-helix (orange), and the client-binding C-terminal domain (CTD; red). **(b)** Schematic representation of full-length TH and variants**.** TH is composed of the regulatory domain (RD) that contains the N-terminal tail (magenta) and an ACT domain (dark blue), followed by the catalytic (light blue) and oligomerization domains (green).

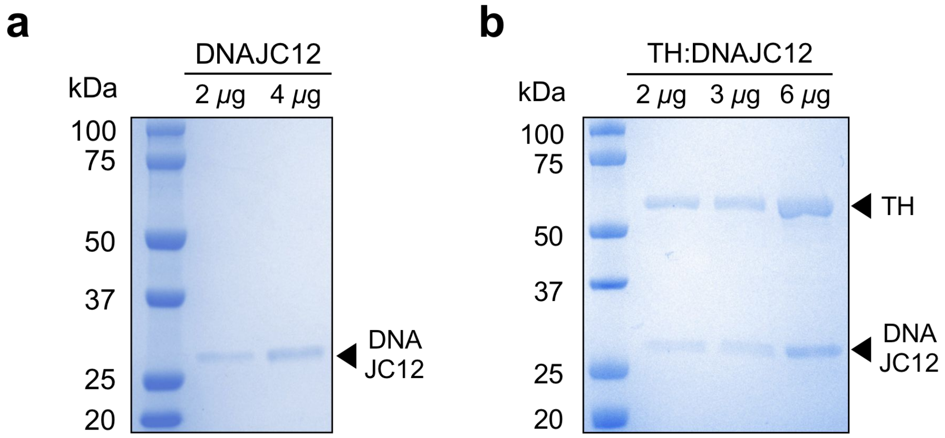

Supplementary Figure 6. SDS-PAGE profiles of DNAJC12 and TH:DNAJC12 complex.

(a) SDS-PAGE of purified recombinant DNAJC12. Pure DNAJC12 (2 or 4 μg) presents a higher apparent molecular mass in SDS-PAGE (~27 kDa; theoretical 23.4 kDa). **(b)** SDS-PAGE analysis of the TH:DNAJC12 complex. SDS-PAGE of the SEC purified complex (2, 3 or 6 μg protein), showing the co-elution of DNAJC12 (~27 kDa) and TH (~56 kDa). Uncropped gels are provided at the end of the Supplementary Information file.

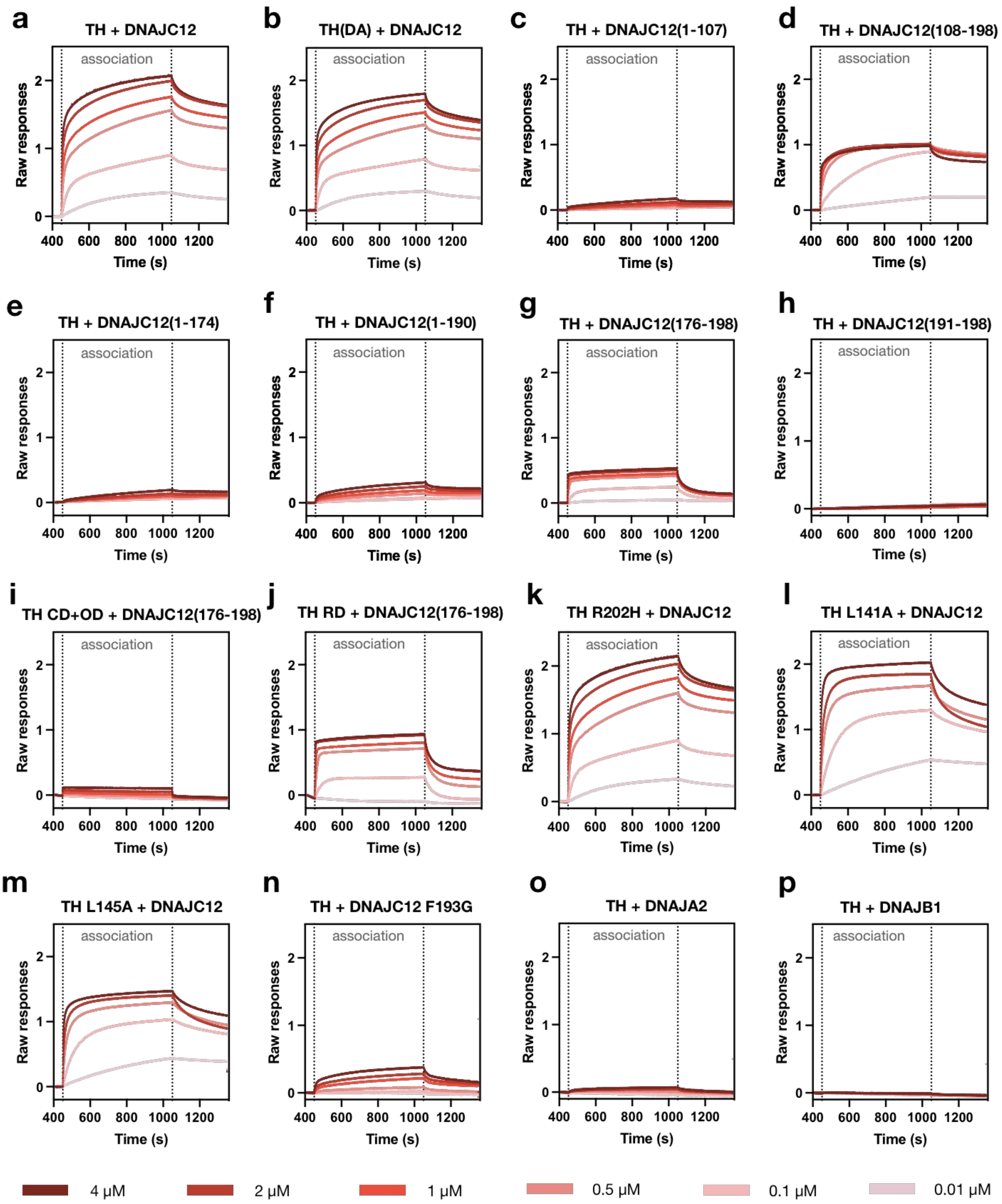

Supplementary Figure 7. BLI binding traces for the interactions investigated in this study.

After 30 s of soaking, 300 s of loading and 120 s of re-equilibration, the binding responses at varying concentrations of either wildtype or the indicated DNAJC12 variants (0.01, 0.1, 0.5, 1, 2, and 4 μM) to either wildtype or the indicated TH variants, were measured during a 600 s association phase (from 450 s to 1050 s) before the final dissociation phase. Steady state analysis was performed using the binding responses recorded in the final 10 s of the association phase. Source data are provided as a Source Data file.

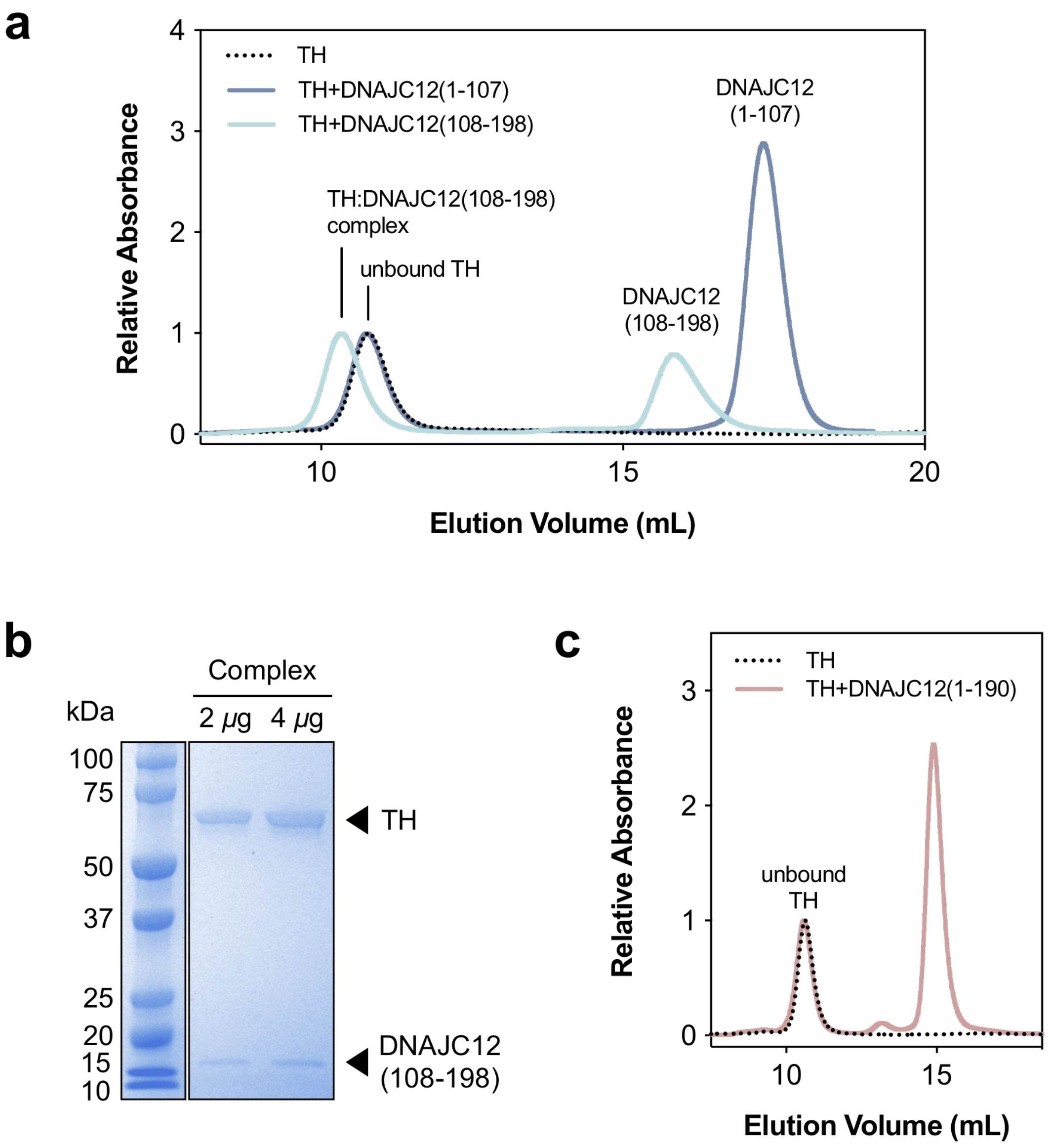

Supplementary Figure 8. Interaction analyses of truncated variants of DNAJC12 to TH.

(a) SEC analyses on a Superdex™ 200 Increase 10/300 GL column of TH with and without DNAJC12(1-107) or DNAJC12(108-198). The complexes are formed with a 1:16 TH (tetramer):DNAJC12 (monomer) ratio. Addition of DNAJC12(108-198) (light blue), but not DNAJC12(1-107) (dark blue), shifts the elution of TH (stippled line) to an earlier volume, indicating binding of the C-terminal section to TH. **(b)** SDS-PAGE of the collected protein samples eluting in the putative complex peak (in **a**), showing the co-elution of TH and DNAJC12(108-198) (~10.5 kDa) in this peak. **(c)** SEC analyses as in (**a**), of TH alone (stippled line) and in the presence of DNAJC12(1-190) (red), showing that the removal of the evolutionarily conserved C-terminal region in DNAJC12 abolishes its ability to bind to TH. Source data are provided as a Source Data file. Uncropped gels are provided at the end of the Supplementary Information file.

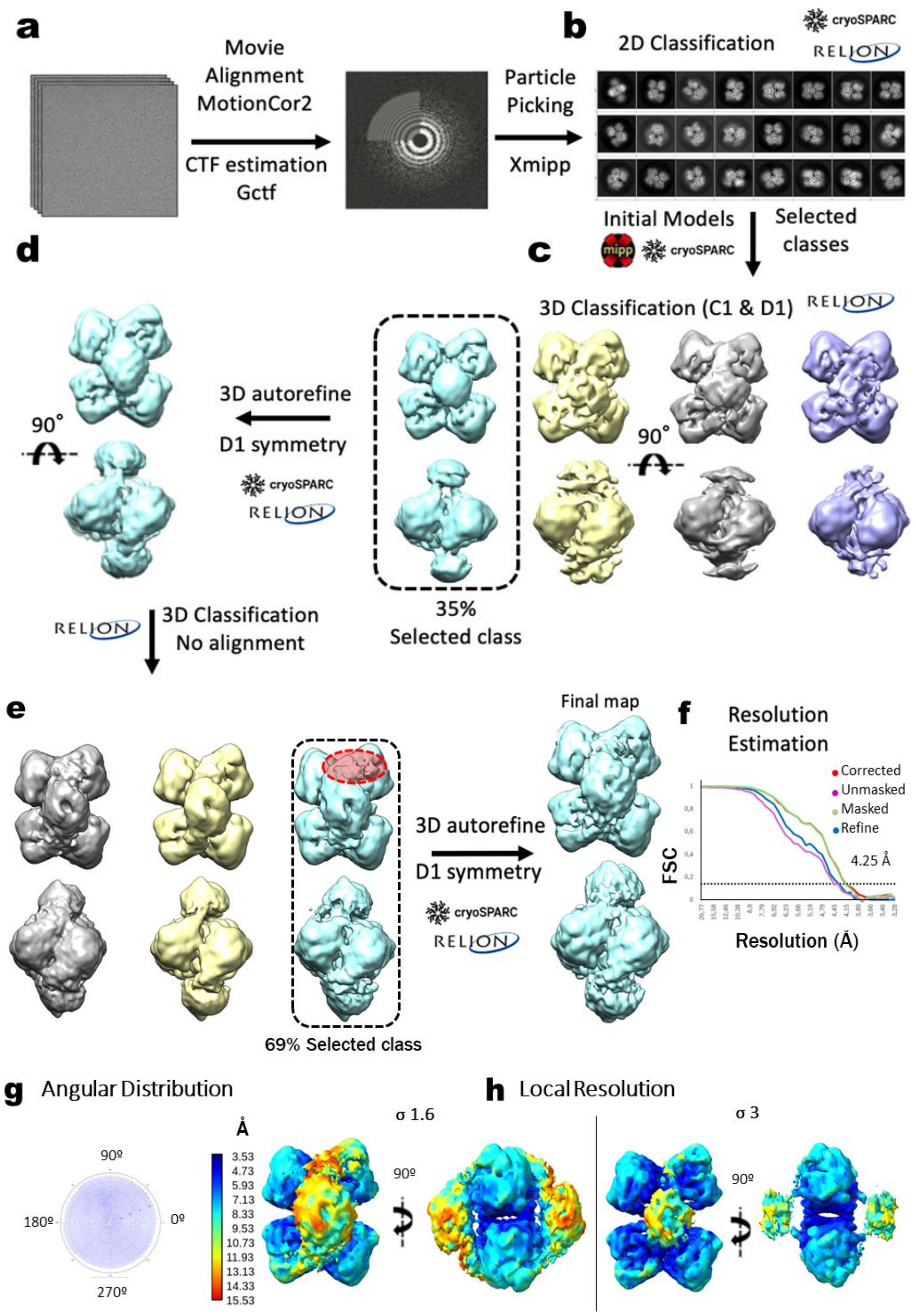

Supplementary Figure 9. Workflow of the 3D reconstruction procedure followed for the TH:DNAJC12 complex.

(a) Movies were acquired using a 300 kV Titan Krios electron microscope. Alignment and Contrast Transfer Function (CTF) calculations were carried out to correct for aberrations introduced by the microscope. **(b)** 2D classification of the collected particles was performed, showing different orientations, which were then used to obtain an initial low-resolution model without imposing any symmetry. **(c)** Initial 3D classification was conducted to align the particles, both with and without imposing symmetry, in order to select the best particles for further processing. **(d)** Refinement of the selected class was performed to obtain the best alignments. **(e)** 3D classification without alignment was carried out to separate those particles showing DNAJC12 bound to the TH and overcome any flexibility in the sample. **(f)** Refinement was performed to obtain the final map. Resolution estimation was conducted using the gold-standard Fourier Shell Correlation (FSC) criterion, with a cutoff of 0.143. **(g)** Angular distribution from the final refinement was evaluated. **(h)** Estimation of the local resolution was performed to assess the quality of the final map.

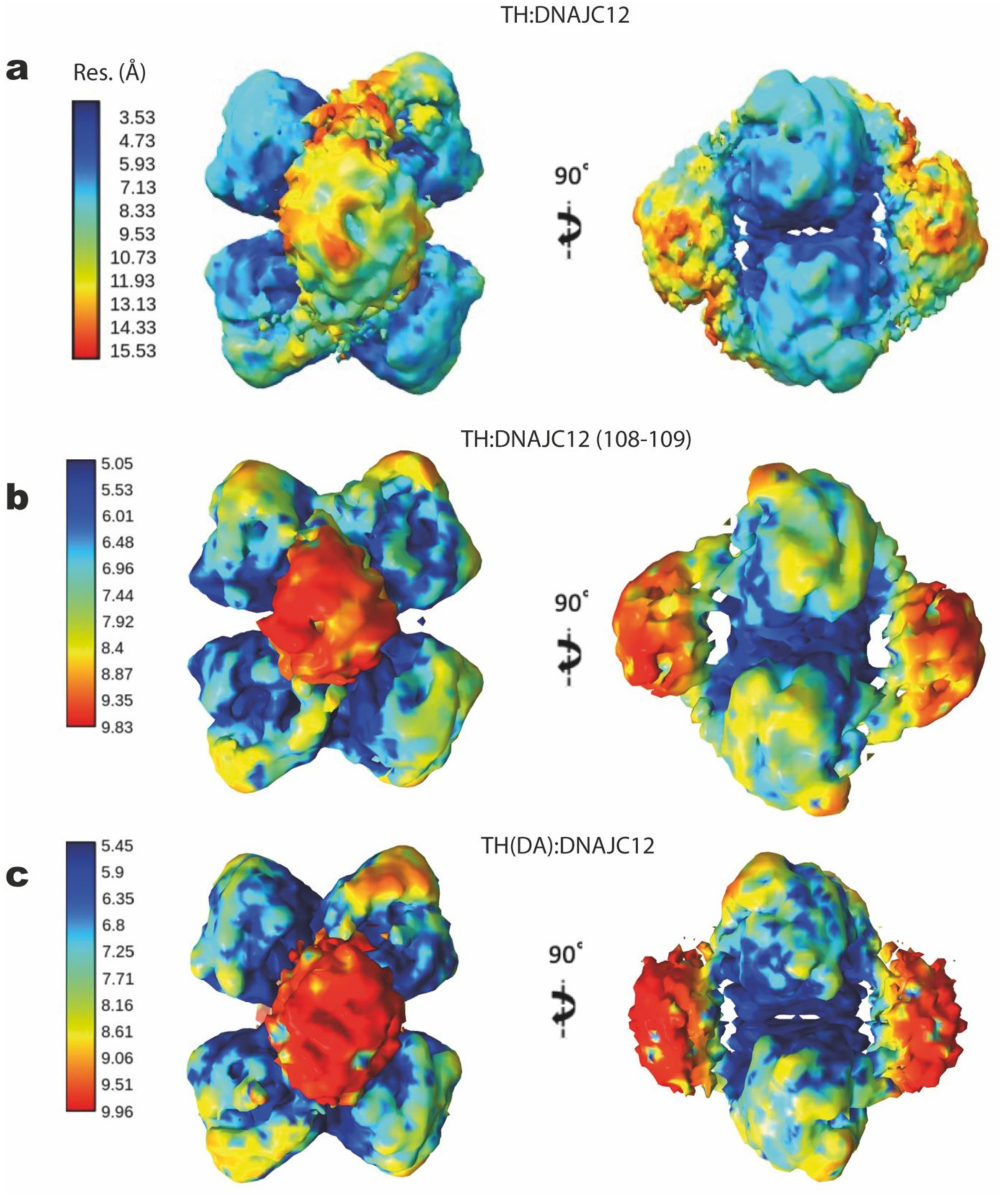

Supplementary Figure 10. Anisotropy in the resolution of the different 3D reconstructions obtained in this work.

Local resolution for the complexes (**a**) TH:DNAJC12, (**b**) TH:DNAJC12(108-198) and (**c**) TH(DA):DNAJC12 was calculated by MonoRes using the half maps of the final volumes.

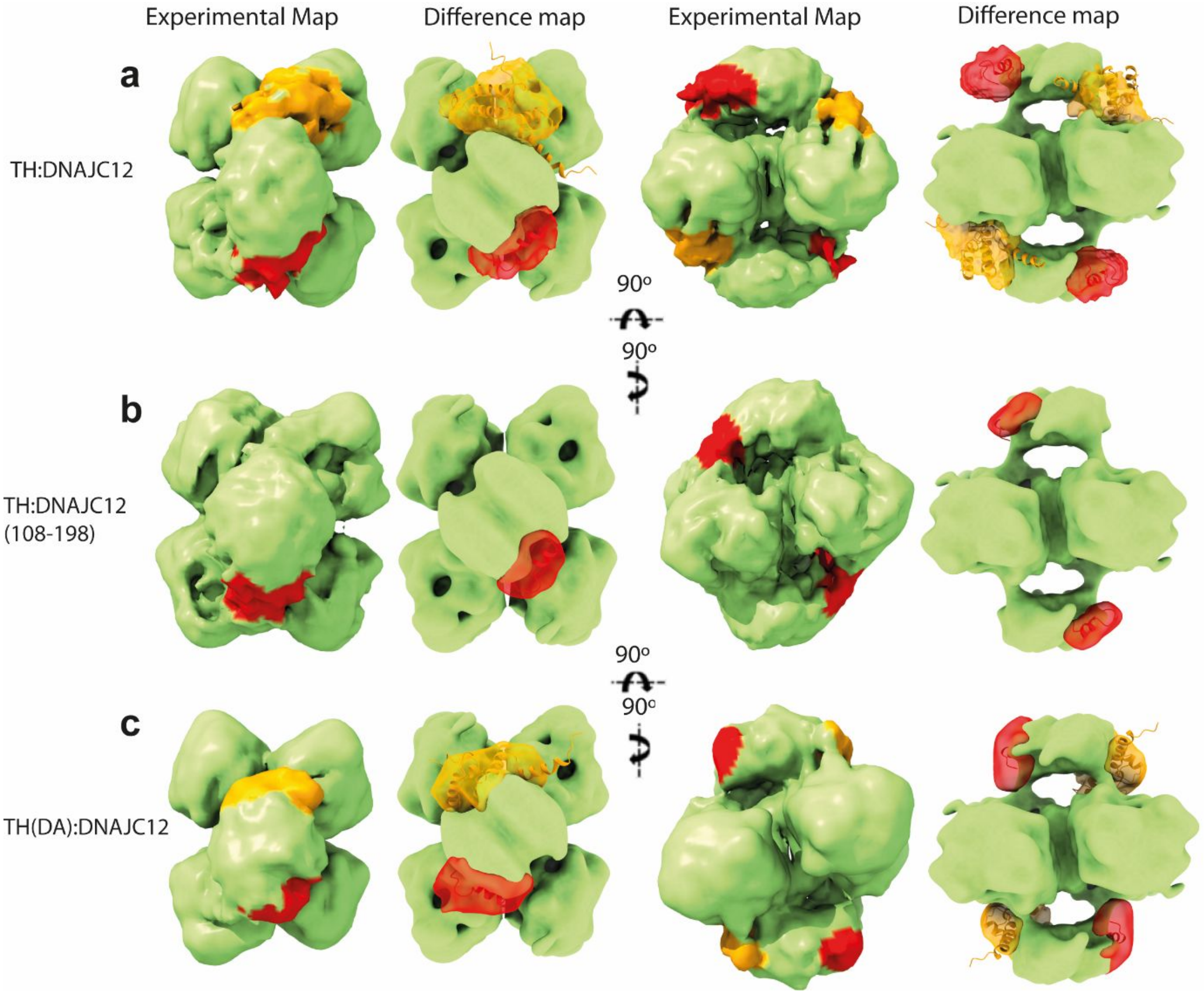

Supplementary Figure 11. Difference maps of TH:DNAJC12, TH:DNAJC12 (108-198) and TH(DA):DNAJC12 subtracted from apo-TH.

Two views, the frontal and lateral, of the experimental maps of the complexes (**a**) TH:DNAJC12, (**b**) TH(DA):DNAJC12, and (**c**) TH:DNAJC12 (108-198) are displayed. Subtracting the density of apo-TH from these maps reveals extra densities (colored in orange and red). Orange regions correspond to the J domain and red regions to the C-terminal domain of the DNAJC12 protein. Additionally, the atomic structures of both DNAJC12 domains are fitted into these extra densities. A slight rotation of the regulatory domains (RD) can be seen in the frontal view of the unsubtracted maps compared with apo-TH.

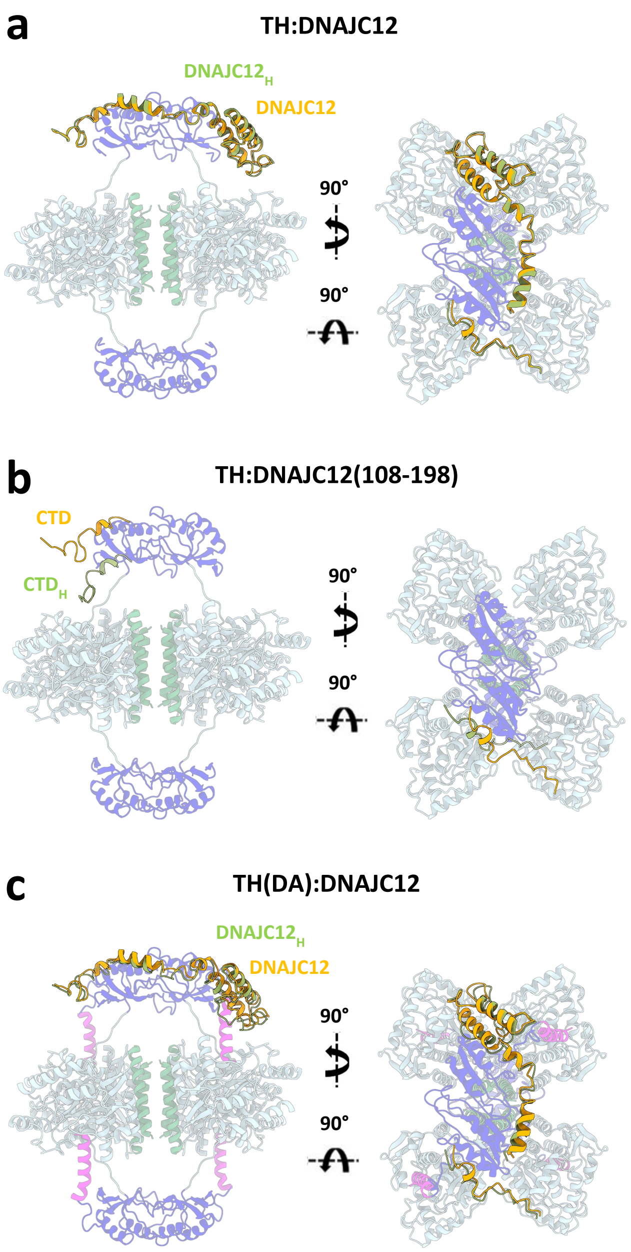

Supplementary Figure 12. XL-MS- and cryoEM-guided docking for TH:DNAJC12, TH:DNAJC12(108-198) and TH(DA):DNAJC12 complexes generated using HADDOCK 2.4.

HADDOCK models for TH:DNAJC12 **(a)**, TH:DNAJC12 (108-198) **(b)** and TH(DA):DNAJC12 **(c)** (denoted with subscript H and colored in green) were superimposed on previously obtained structural models based on the cryo-EM data (colored in orange). To highlight the position of the different DNAJC12 domains, the TH structure has been faded out. Note that HADDOCK-based docking models closely resemble our structural model.

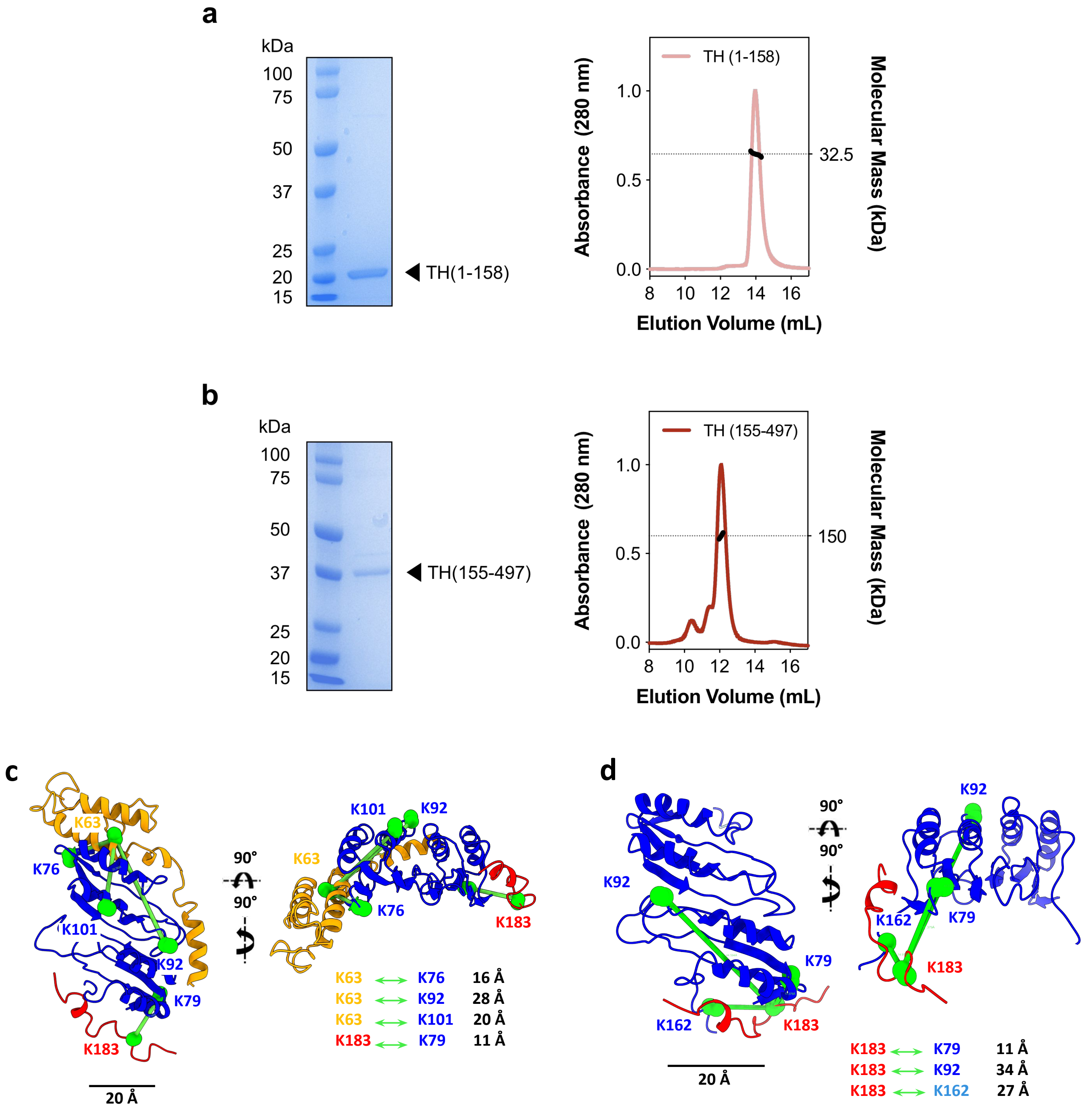

Supplementary Figure 13. Characterization of the oligomeric distribution of isolated TH domains.

(a) SDS-PAGE (left) of the purified regulatory domain (RD; residues 1-158), showing the correct theoretical monomeric size of the RD (~17 kDa). Analysis by SEC-MALS (right) confirmed that the isolated RD was dimeric (32.5 ± 0.17 kDa). **(b)** SDS-PAGE analysis (left) of the purified catalytic and oligomerization domains (CD+OD; residues 155-497) also showing the correct monomeric size of the CD+OD (~38 kDa). Subsequent SEC-MALS analysis determined that the CD+OD was tetrameric (150 ± 0.099 kDa). Source data are provided as a Source Data file. Uncropped gels are provided at the end of the Supplementary Information file. **(c)** and **(d)** show two views of TH RD:DNAJC12 and TH RD:CTD models based on the interprotein crosslinks obtained by XL-MS for both complexes. The TH RDs are colored in dark blue while the AlphaFold model of DNAJC12 has the JD in orange and the CTD in red. The displayed crosslinks (highlighted in gray in Supplementary Table 2) are shown only for one side of the different TH complexes and are shown in green. Note that the distances of all crosslinks are also depicted.

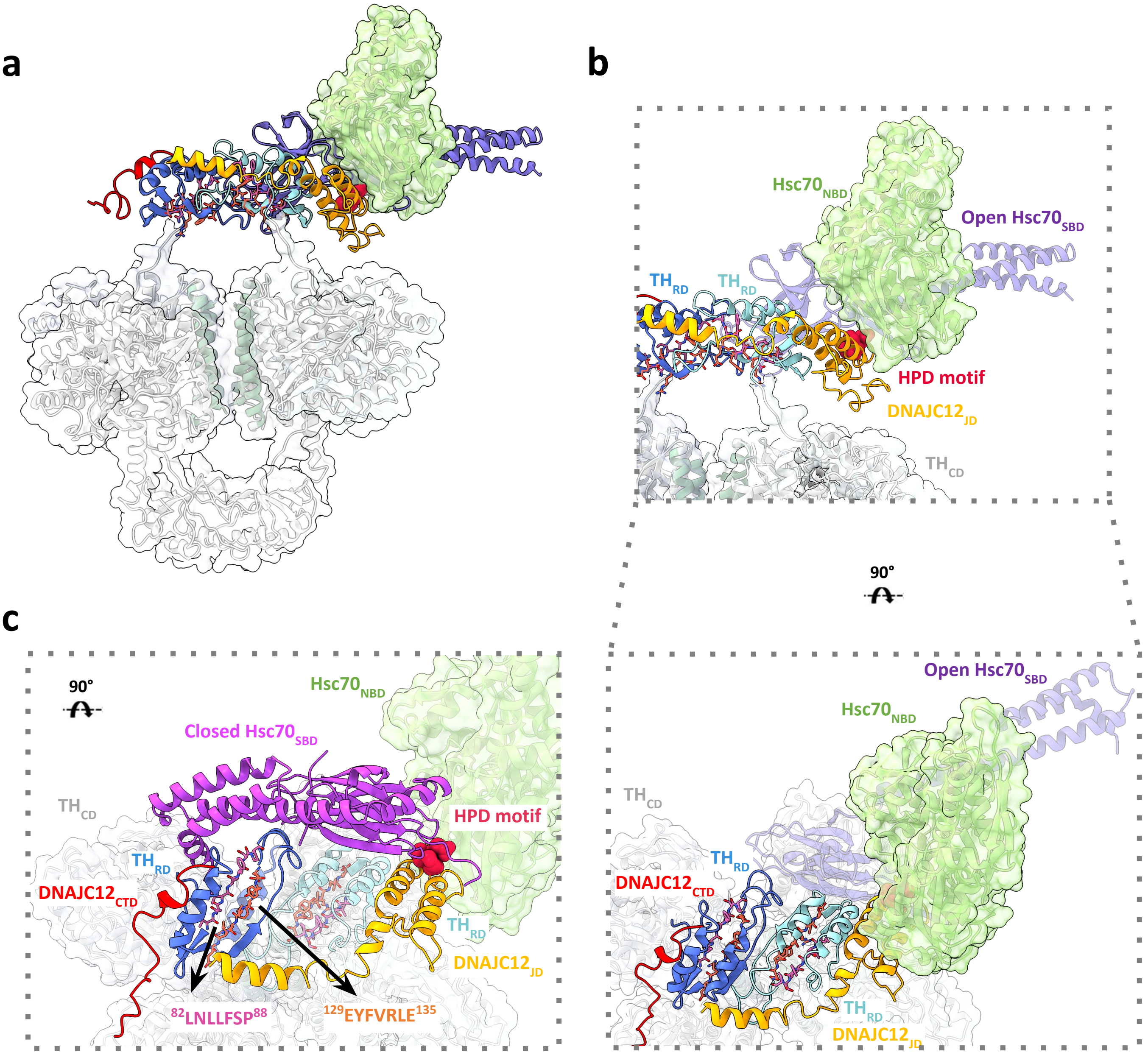

Supplementary Figure 14. Model of the interaction of the TH:DNAJC12 complex with Hsc70, through the DNAJC12 HPD motif.

(a) A combined ribbon and surface model of the TH:DNAJC12 complex is superimposed on the experimental model of the ATP-bound Hsp70 of *Escherichia coli* (DnaK), in complex with JD (PDB 5NRO)[7]. **(b)** Two views of DNAJC12, with JD in gold, HDP motif as red spheres, and CTD in red (linker-helix is not included), binds to TH (PDB 6ZVP)[8] by embracing the RD dimer (RD subunits in cyan and light blue). The NBD of Hsp70 is shown in surface representation (green) and the SBD, in open conformation, as purple ribbon. The JDs from both models (TH:DNAJC12 complex and PDB 5NRO) have been aligned using ChimeraX software, and the JD from PDB 5NRO has been excluded after structural alignment for clarity. **(c)** Superposition of the ADP-bound closed structure of the Hsp70 SBD (PDB 1DKX)[9] on the model shown in (**b**) places the SBD in the vicinity of one of the TH-RDs (light blue), with β-strand regions ^{82}LNLLLFSP^{88} (pink sticks) and ^{129}EYFVRLE^{135} (orange sticks), predicted to bind to the Hsp70 family by Limbo[10] and ChaperISM[11] algorithms. Region 82-88 is also predicted by TANGO[12,13] to be highly predisposed to form aggregating intermolecular cross-β interactions (see main text). The model aligns well with a role for the TH:DNAJC12 complex, where DNAJC12 stabilizes TH and ultimately presents it to Hsc70. Hsc70 simultaneously recognizes TH as a client and undergoes ATP hydrolysis facilitated by the TH-bound DNAJC12. See also Supplementary movie 2.

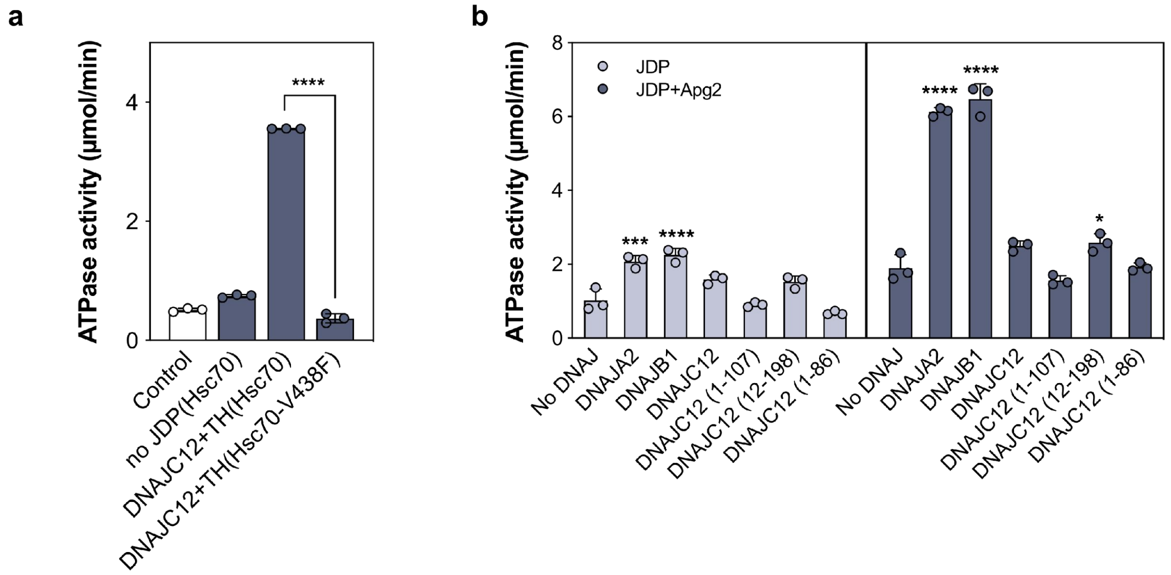

Supplementary Figure 15. The stimulation of ATPase activity of Hsc70 (a) compared with that with variant Hsc70-V438F, and (b) by truncated DNAJC12 variants in the absence or presence of Apg2.

(a) ATPase activity of Hsc70-V438F which has low affinity for substrates, was measured in the presence of DNAJC12 and TH and compared to that of wildtype Hsc70. The data represent the mean ± SD for n=3 independent experiments. The ATPase activity of Hsc70 or Hsc70-V438F recorded with Apg2 and DNAJC12 with TH were compared by Student's t-test (****p<0.0001). **(b)** The ability of truncated DNAJC12 variants to stimulate Hsc70 ATPase activity was compared to that of DNAJA2 and DNAJB1 (1µM JDP concentration). The data represent the mean ± SD for n=3 independent experiments. The ATPase activity of Hsc70 recorded with the different JDPs was compared to their respective controls (either with (dark blue) or without Apg2 (light purple)), that only have Hsc70 and do not contain any JDP, by one-way ANOVA and Tukey's post-hoc test (*p=0.0245; ***p=0.0001; ****p<0.0001). A small statistically-significant increase in Hsc70 ATPase activity was recorded in the presence of the DNAJC12 variant lacking the N-terminal tail, DNAJC12(12-198). However, using the same multiple comparison analysis, there was no significant difference found in the Hsc70 ATPase activities recorded in the presence of full-length DNAJC12 or DNAJC12(12-198). Source data are provided as a Source Data file.

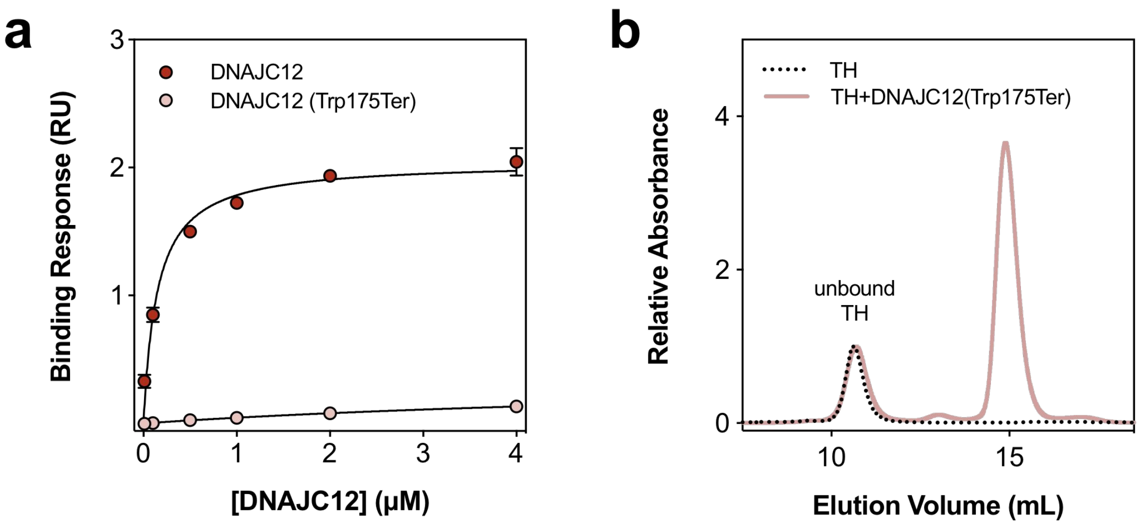

Supplementary Figure 16. Binding analyses of the disease-associated variant DNAJC12-W175Ter to TH.

(a) Binding analyses of DNAJC12-W175Ter to TH by BLI. Binding responses to immobilized TH were recorded at 0.01, 0.1, 0.5, 1, 2 and 4 μM DNAJC12, full-length (red) or DNAJC12-W175Ter (pink). Unlike the control samples with TH and full-length DNAJC12, low binding response was measured for the disease variant. The binding responses are presented as mean ± 95% CI for 3 independent samples, and the fitting to a non-linear regression curve provided K_D values of 148 ± 18 nM for full-length DNAJC12, and 7674 ± 1237 nM for DNAJC12-W175Ter. **(b)** SEC analyses of samples containing a 1:16 molar ratio of TH tetramer:DNAJC12-W175Ter monomer compared with a sample containing TH alone**.** Addition of the DNAJC12-W175Ter (pink line), does not shift the elution of TH (black stippled line) to an earlier volume, indicating that the two proteins do not form a complex. Source data are provided as a Source Data file.

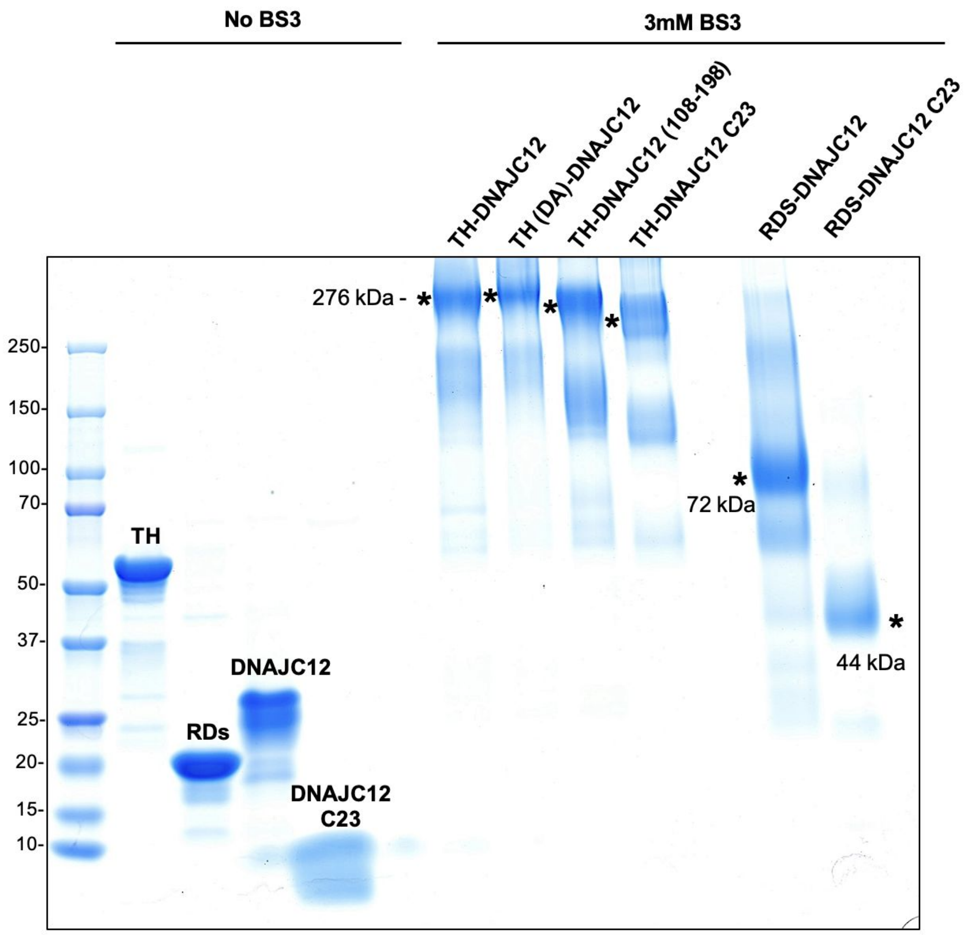

Supplementary Figure 17. Crosslinked samples of the TH:DNAJC12 complexes analyzed by XL-MS.

SDS-PAGE of the different crosslinked TH:DNAJC12 complexes and their truncated variants using BS3 crosslinker. Non-crosslinked protein controls are shown on the left. Bands marked with asterisks indicate those extracted from the gel for the XL-MS analysis, along with their approximate molecular masses.

Data collection	TH:DNAJC12<br>EMD-18047	TH:DNAJC12(108-198)<br>EMD-18058	TH(DA):DNAJC12<br>EMD-18289
**Microscope**	FEI Titan Krios	FEI Titan Krios	FEI Titan Krios
**Voltage (keV)**	300	300	300
**Detector**	Gatan K3	Gatan K3	Gatan K3
**Nominal magnification**	105,000x	105,000x	105,000x
**Pixel size (Å)**	0.85	0.85	0.85
**Defocus range (µm)**	-1.0 to -2.6	-1.0 to -2.6	-1.0 to -2.6
**Exposure time (s)**	1	1	1
**Electron dose (e^-/Å^2)**	40-42	40-42	40-42
**Frames**	40	40	40
**Dose/frame (e^-/Å^2)**	1	1	1
**Movies (no.)**	21,430	18,329	12,826
**Initial particles (no.)**	6,534,161	1,283,996	2,474,620
**Final particles (no.)**	294,287	128,537	46,555
**Final resolution (Å)**	D1	D1	D1
	4.25	5	5.7

Supplementary Table 1. Parameters for cryoEM data collection.

		TH	TH (DA)	RDs (TH)
DNAJC12	J-domain	^{62}A**K**EILTNEESR^{72}–^{17}AVSELDA**K**QAEAIMSPR^{33}	^{62}A**K**EILTNEESR^{72}–^{17}AVSELDA**K**QAEAIMSPR^{33}	^{62}A**K**EILTNEESR^{72}–^{99}AV**K**VFETFEAK^{109} (*)
		^{62}A**K**EILTNEESR^{72}–^{90}AT**K**PSALSR^{98} (*)	^{62}A**K**EILTNEESR^{72}–^{90}AT**K**PSALSR^{98} (*)	^{62}A**K**EILTNEESR^{72}–^{90}AT**K**PSALSR^{98} (*)
		^{62}A**K**EILTNEESR^{72}–^{1}GPTPDATTPQA**K**^{12}	^{62}A**K**EILTNEESR^{72}–^{99}AV**K**VFETFEAK^{109} (*)	^{62}A**K**EILTNEESR^{72}–^{17}AVSELDA**K**QAEAIMSPR^{33}
		^{62}A**K**EILTNEESR^{72}–^{157}SPAGP**K**VPWFPR^{168}	^{62}A**K**EILTNEESR^{72}–^{204}**K**LIAEIAFQYR^{214}	^{62}A**K**EILTNEESR^{72}–^{1}GPTPDATTPQA**K**^{12}
			^{81}SQMSMPFQQWEALNDSV**K**TSMHWVVR^{106}–^{157}SPAGP**K**VPWFPR^{168} (*)	^{62}A**K**EILTNEESR^{72}–^{50}EAAVAAAAAAVPSEPGDPLEAVAFEE**K**EGK^{79} (*)
			^{59}LQKA**K**EILTNEESR^{72}–^{16}RAVSELDA**K**QAEAIMSPR^{33}	^{2}**D**AILNYR^{8}-^{17}AVSELDA**K**QAEAIMSPR^{33}
			^{62}A**K**EILTNEESR^{72}–^{222}VEYTAEEIATW**K**EVYTTLK^{240}	^{2}**D**AILNYR^{8}-^{90}AT**K**PSALSR^{98}
			^{62}A**K**EILTNEESR^{72}–^{1}GPTPDATTPQA**K**^{12}	^{52}AVETFQ**K**LQK^{61}-^{17}AVSELDA**K**QAEAIMSPR^{33}
			^{62}A**K**EILTNEESR^{72}–^{157}SPAGP**K**VPWFPR^{168}	
			^{52}AVETFQ**K**LQK^{61}-^{17}AVSELDA**K**QAEAIMSPR^{33}	
			^{52}AVETFQ**K**LQK^{61}-^{90}AT**K**PSALSR^{98} (*)	
			^{52}AVETFQ**K**LQK^{61}–^{157}SPAGP**K**VPWFPR^{168} (*)	
			^{81}SQMSMPFQQWEALNDSV**K**TSMHWVVR^{106}–^{17}AVSELDA**K**QAEAIMSPR^{33}	
			^{59}LQKA**K**EILTNEESR^{72}–^{17}AVSELDA**K**QAEAIMSPR^{33}	
			^{2}**D**AILNYR^{8}–^{157}SPAGP**K**VPWFPR^{168}	
	Linker	^{134}ER**K**^{136}–^{90}AT**K**PSALSR^{98}	^{136}K**K**EELASTAEK^{146}–^{90}AT**K**PSALSR^{98}	^{137}**K**EELASTAEK^{146}–^{99}AV**K**VFETFEAK^{109}
		^{134}ER**K**^{136}–^{17}AVSELDA**K**QAEAIMSPR^{33}	^{109}**K**DLMLEESDK^{118}–^{157}SPAGP**K**VPWFPR^{168}	^{137}**K**EELASTAEK^{146}–^{17}AVSELDA**K**QAEAIMSPR^{33}
		^{134}ER**K**^{136}–^{204}**K**LIAEIAFQYR^{214}	^{119}THTT**K**MENEEBNEQR^{133}–^{90}AT**K**PSALSR^{98}	^{137}**K**EELASTAEK^{146}–^{90}AT**K**PSALSR^{98}
		^{136}K**K**EELASTAEK^{146}–^{90}AT**K**PSALSR^{98}	^{109}**K**DLMLEESDK^{118}–^{90}AT**K**PSALSR^{98}	^{137}**K**EELASTAEK^{146}–^{1}GPTPDATTPQA**K**^{12}
		^{147}TEQ**K**^{150}–^{234}EVYTTL**K**^{240}	^{109}**K**DLMLEESDK^{118}–^{17}AVSELDA**K**QAEAIMSPR^{33}	^{138}EELA**S**TAEKTEQK^{150}–^{17}AVSELDA**K**QAEAIMSPR^{33}
			^{136}K**K**EELASTAEK^{146}–^{169}**K**VSELDK^{175}	^{138}EELA**S**TAEKTEQK^{150}–^{1}GPTPDATTPQA**K**^{12}
			^{137}**K**EELASTAEK^{146}–^{99}AV**K**VFETFEAK^{109}	^{147}TEQ**K**^{150}–^{1}GPTPDATTPQA**K**^{12}
			^{137}**K**EELASTAEK^{146}–^{04}**K**LIAEIAFQYR^{214}	^{147}TEQ**K**^{150}–^{90}AT**K**PSALSR^{98}
			^{137}**K**EELASTAEK^{146}–^{17}AVSELDA**K**QAEAIMSPR^{33}	^{108}DLMLEESD**K**THTTK^{123}–^{90}AT**K**PSALSR^{98}
				^{109}**K**DLMLEESDK^{118}–^{47}**K**ER^{49}
				^{109}**K**DLMLEESDK^{118}–^{90}AT**K**PSALSR^{98}
	CTD	^{181}WS**K**DAPSELLR^{191}–^{47}**K**ER^{49}	^{181}WS**K**DAPSELLR^{191}–^{77}EG**K**AMLNLLFSPR^{89} (*)	^{181}WS**K**DAPSELLR^{191}–^{77}EG**K**AMLNLLFSPR^{89} (*)
		^{181}WS**K**DAPSELLR^{191}–^{77}EG**K**AMLNLLFSPR^{89} (*)	^{181}WS**K**DAPSELLR^{191}–^{17}AVSELDA**K**QAEAIMSPR^{33}	^{181}WS**K**DAPSELLR^{191}–^{17}AVSELDA**K**QAEAIMSPR^{33}
		^{181}WS**K**DAPSELLR^{191}–^{169}**K**VSELDK^{175} (*)	^{181}WS**K**DAPSELLR^{191}–^{157}SPAGP**K**VPWFPR^{168} (*)	^{181}WS**K**DAPSELLR^{191}–^{1}GPTPDATTPQA**K**^{12}
		^{181}WS**K**DAPSELLR^{191}–^{157}SPAGP**K**VPWFPR^{168} (*)	^{181}WS**K**DAPSELLR^{191}–^{169}**K**VSELDK^{175} (*)	^{181}WS**K**DAPSELLR^{191}–^{90}AT**K**PSALSR^{98}
		^{181}WS**K**DAPSELLR^{191}–^{50}EAAVAAAAAAVPSEPGDPLEAVAFEE**K**EGK^{79}	^{181}WS**K**DAPSELLR^{191}–^{17}AVSELDA**K**QAEAIMSPR^{33}	
		^{181}WS**K**DAPSELLR^{191}–^{1}GPTPDATTPQA**K**^{12}	^{181}WS**K**DAPSELLR^{191}–^{204}**K**LIAEIAFQYR^{214}	
		^{181}WS**K**DAPSELLR^{191}–^{278}FL**K**ER^{282}	^{181}WS**K**DAPSELLR^{191}–^{90}AT**K**PSALSR^{98} (*)	
		^{181}WS**K**DAPSELLR^{191}–^{17}AVSELDA**K**QAEAIMSPR^{33}	^{181}WS**K**DAPSELLR^{191}–^{39}Q**S**LIEDAR^{46}	
		^{184}DAP**S**ELLR^{191}–^{157}SPAGP**K**^{162} (*)	^{181}WS**K**DAPSELLR^{191}–^{1}GPTPDATTPQA**K**^{12}	
		^{192}**K**FR^{194}–^{278}FL**K**ER^{282}	^{192}**K**FR^{194}–^{278}FL**K**ER^{282}	
		^{192}**K**FR^{194}–^{157}SPAGP**K**VPWFPR^{168} (*)		
		^{192}**K**FR^{194}–^{169}**K**VSELDK^{175}		
		^{136}K**K**EELASTAEK^{146}–^{90}AT**K**PSALSR^{98}		
		^{136}K**K**EELASTAEK^{146}–^{169}**K**VSELDK^{175}		
		^{136}K**K**EELASTAEK^{146}–^{157}SPAGP**K**VPWFPR^{168}		
		^{136}K**K**EELASTAEK^{146}–^{204}**K**LIAEIAFQYR^{214}		
		^{136}K**K**EELASTAEK^{146}–^{222}VEYTAEEIATW**K**EVYTTLK^{240}		
		^{136}K**K**EELASTAEK^{146}–^{47}**K**ER^{49}		

DNAJC12 (108-198)		^{136}K**K**EELASTAEK^{146}–<br>^{16}RAVSELDA**K**QAEAIMSPR^{33}		
		^{138}EELA**S**TAEKTEQK^{150}–<br>^{17}AVSELDA**K**QAEAIMSPR^{33}		
		^{138}EELA**S**TAEKTEQK^{150}—^{90}AT**K**PSALSR^{98}		
		^{138}EELA**S**TAEKTEQK^{150}—^{157}SPAGP**K**VPWFPR^{168}		
		^{138}EELA**S**TAEKTEQK^{150}—^{204}**K**LIAEIAFQYR^{214}		
		^{109}**K**DLMLEESDK^{118}–^{90}AT**K**PSALSR^{98}		
		^{158}SVSPQN**S**DSSGFADVNGWHLR^{178}–<br>^{157}SPAGP**K**VPWFPR^{168}		
		^{119}THTT**K**MENEEBNEQR^{133}–^{90}AT**K**PSALSR^{98}		
		^{119}THTT**K**MENEEBNEQR^{133}–<br>^{17}AVSELDA**K**QAEAIMSPR^{33}		
		^{147}TEQ**K**^{150}–^{90}AT**K**PSALSR^{98}		
	CTD	^{181}WS**K**DAPSELLR^{191}–^{77}EG**K**AMLNLLFSPR^{89} **(*)**		
		^{181}WS**K**DAPSELLR^{191}–^{157}SPAGP**K**VPWFPR^{168} **(*)**		
		^{181}WS**K**DAPSELLR^{191}–^{1}GPTPDATTPQA**K**^{12}		
		^{181}WS**K**DAPSELLR^{191}–<br>^{17}AVSELDA**K**QAEAIMSPR^{33}		
		^{181}WS**K**DAPSELLR^{191}–<br>^{222}VEYTAEEIATW**K**EVYTTLK^{240}		
		^{181}WS**K**DAPSELLR^{191}–<br>^{459}FDP**Y**TLAIDVLDSPQAVR^{476}		
		^{181}WS**K**DAPSELLR^{191}–^{204}**K**LIAEIAFQYR^{214}		
		^{181}WS**K**DAPSELLR^{191}–^{90}AT**K**PSALSR^{98} **(*)**		
		^{192}**K**FR^{194}–^{157}SPAGP**K**VPWFPR^{168}		
		^{181}WS**K**DAPSELLR^{191}–<br>^{381}QNGEV**K**AYGAGLLSSYGELLHCLSEEPEIR^{410}		
		^{181}WS**K**DAPSELLR^{191}–^{169}**K**VSELDK^{175} **(*)**		
		^{151}EP**K**PLEKSVSPQNSDSSGFADVNGWHLR^{178}–<br>^{17}AVSELDA**K**QAEAIMSPR^{33}		
DNAJC12 (176-198)	CTD			^{181}WS**K**DAPSELLR^{191}–^{77}EG**K**AMLNLLFSPR^{89} **(*)**
				^{181}WS**K**DAPSELLR^{191}–^{157}SPAGP**K**VPWFPR^{168}**(*)**
				^{181}WS**K**DAPSELLR^{191}–^{90}AT**K**PSALSR^{98} **(*)**
				^{181}WS**K**DAPSELLR^{191}–^{17}AVSELDA**K**QAEAIMSPR^{33}

Supplementary Table 2. XL-MS analysis of TH:DNAJC12, TH:DNAJC12(108-198), TH:DNAJC12(176-198), TH(DA):DNAJC12 and TH-RD:DNAJC12 complexes.

Peptides identified by XL-MS corresponding to interprotein crosslinks are listed. For every crosslink, the first peptide belongs to DNAJC12 and the second to TH. The peptide sequence and the positions of the first and last residues of each peptide are indicated, while the crosslinking sites are highlighted in bold. The peptide pairs are organized by their location within DNAJC12 (either the J-domain (JD), linker or the C-terminal domain (CTD) and whether they were found in the full-length TH samples with and without dopamine (DA) or in the truncated TH form containing only the regulatory domain (RD). Note that crosslinks highlighted in gray are those corresponding to structured regions of TH and DNAJC12 used for the structural analysis and represented in Fig. 5.

			Max. distance ChimeraX (Å)	
DNAJC12	^{62}AKEILTNEESR^{72}	^{90}ATKPSALSR^{98}	28.046	TH WT
	^{181}WSKDAPSELLR^{191}	^{77}EGKAMLNLLFSPR^{89}	11.378	
	^{181}WSKDAPSELLR^{191}	^{169}KVSELDK^{175}	33.938	
	^{181}WSKDAPSELLR^{191}	^{157}SPAGPKVPWFPR^{168}	27.332	
	^{184}DAPSELLR^{191}	^{157}SPAGPK^{162}	23.879	
	^{192}KFR^{194}	^{157}SPAGPKVPWFPR^{168}	26.661	
	^{52}AVETFQKLQK^{61}	^{90}ATKPSALSR^{98}	28.599	TH (DA)
	^{52}AVETFQKLQK^{61}	^{157}SPAGPKVPWFPR^{168}	21.395	
	^{62}AKEILTNEESR^{72}	^{90}ATKPSALSR^{98}	28.046	
	^{62}AKEILTNEESR^{72}	^{99}AVKVFETFEAK^{109}	20.489	
	^{81}SQMSMPFQQWEALNDSVKTSMHWVVR^{107}	^{157}SPAGPKVPWFPR^{168}	32.259	
	^{181}WSKDAPSELLR^{191}	^{77}EGKAMLNLLFSPR^{89}	11.378	
	^{181}WSKDAPSELLR^{191}	^{169}KVSELDK^{175}	33.938	
	^{181}WSKDAPSELLR^{191}	^{157}SPAGPKVPWFPR^{168}	27.322	
	^{181}WSKDAPSELLR^{191}	^{90}ATKPSALSR^{98}	34.005	
	^{62}AKEILTNEESR^{72}	^{99}AVKVFETFEAK^{109}	20.489	RDs (TH)
	^{62}AKEILTNEESR^{72}	^{90}ATKPSALSR^{98}	28.046	
	^{62}AKEILTNEESR^{72}	^{50}EAAVAAAAAAVPSEPGDPLEAVAFEE KEGK^{79}	16.065	
	^{181}WSKDAPSELLR^{191}	^{77}EGKAMLNLLFSPR^{89}	11.378	
DNAJC12 CTD	^{181}WSKDAPSELLR^{191}	^{77}EGKAMLNLLFSPR^{89}	11.378	TH WT
	^{181}WSKDAPSELLR^{191}	^{157}SPAGPKVPWFPR^{168}	27.322	
	^{181}WSKDAPSELLR^{191}	^{169}KVSELDK^{175}	33.938	
	^{181}WSKDAPSELLR^{191}	^{90}ATKPSALSR^{98}	34.005	
DNAJC12 C23	^{181}WSKDAPSELLR^{191}	^{77}EGKAMLNLLFSPR^{89}	11.378	
	^{181}WSKDAPSELLR^{191}	^{157}SPAGPKVPWFPR^{168}	27.322	
	^{181}WSKDAPSELLR^{191}	^{90}ATKPSALSR^{98}	34.005	

Supplementary Table 3. Comparison of the XL-MS analysis using MeroX and XlinkX search engines.

The table presents the interpeptide cross-links used for the structural analysis determined using both MeroX and XlinkX, and their respective distance measured by ChimeraX. Color code follows crosslinks from MeroX (blue), XlinkX (yellow) and both (green). Over 75% of the crosslinks are shared among both analyses. On the left side of the table, the peptides from DNAJC12 (either full-length or truncated variants) are listed alongside the corresponding peptides from TH on the right side (either full-length, regulatory domains (RDs), or in the presence of dopamine) where the interpeptide crosslinks were identified**.**

			MAX.DISTANCE (Å)				
			TH monomer 1	TH monomer 2	TH monomer 3	TH monomer 4	
DNAJC12	^{62}A**K**EILTNEESR^{72}	^{90}AT**K**PSALSR^{98}	28.046	112.294	109.943	31.83	TH WT
DNAJC12	^{181}WS**K**DAPSELLR^{191}	^{77}EG**K**AMLNLLFSPR^{89}	11.378	99.574	87.483	56.323	TH WT
DNAJC12	^{181}WS**K**DAPSELLR^{191}	^{169}**K**VSELDK^{175}	33.938	80.984	56.357	64.684	TH WT
DNAJC12	^{184}DAP**S**ELLR^{191}	^{157}SPAGP**K**^{162}	23.879	90.864	78.085	51.67	TH WT
DNAJC12	^{192}**K**FR^{194}	^{157}SPAGP**K**VPWFPR^{168}	26.661	94.343	86.339	51.596	TH WT
DNAJC12	^{62}A**K**EILTNEESR^{72}	^{90}AT**K**PSALSR^{98}	28.046	112.294	109.943	31.83	TH (DA)
DNAJC12	^{62}A**K**EILTNEESR^{72}	^{99}AV**K**VFETFEAK^{109}	35.192	106.022	109.772	20.489	TH (DA)
DNAJC12	^{81}SQMSMPFQQWEAL NDSV**K**TSMHWVVR^{107}	^{157}SPAGP**K**VPWFPR^{168}	32.259	94.833	84.172	46.131	TH (DA)
DNAJC12	^{181}WS**K**DAPSELLR^{191}	^{77}EG**K**AMLNLLFSPR^{89}	11.378	99.574	87.483	56.323	TH (DA)
DNAJC12	^{181}WS**K**DAPSELLR^{191}	^{169}**K**VSELDK^{175}	33.938	80.984	56.357	64.684	TH (DA)
DNAJC12	^{181}WS**K**DAPSELLR^{191}	^{157}SPAGP**K**VPWFPR^{168}	27.322	89.732	74.197	49.64	TH (DA)
DNAJC12	^{181}WS**K**DAPSELLR^{191}	^{90}AT**K**PSALSR^{98}	42.684	105.761	107.056	34.005	TH (DA)
DNAJC12	^{62}A**K**EILTNEESR^{72}	^{99}AV**K**VFETFEAK^{109}	35.192	-	-	20.489	RDs (TH)
DNAJC12	^{62}A**K**EILTNEESR^{72}	^{90}AT**K**PSALSR^{98}	28.046	-	-	31.83	RDs (TH)
DNAJC12	^{181}WS**K**DAPSELLR^{191}	^{77}EG**K**AMLNLLFSPR^{89}	11.378	-	-	56.323	RDs (TH)
DNAJC12 CTD	^{181}WS**K**DAPSELLR^{191}	^{77}EG**K**AMLNLLFSPR^{89}	11.378	99.574	87.483	56.323	TH WT
DNAJC12 CTD	^{181}WS**K**DAPSELLR^{191}	^{157}SPAGP**K**VPWFPR^{168}	27.322	89.732	74.197	49.64	TH WT
DNAJC12 CTD	^{181}WS**K**DAPSELLR^{191}	^{90}AT**K**PSALSR^{98}	42.684	105.761	107.056	34.005	TH WT
DNAJC12 C23	^{181}WS**K**DAPSELLR^{191}	^{77}EG**K**AMLNLLFSPR^{89}	11.378	99.574	87.483	56.323	TH WT
DNAJC12 C23	^{181}WS**K**DAPSELLR^{191}	^{157}SPAGP**K**VPWFPR^{168}	27.322	89.732	74.197	49.64	TH WT
DNAJC12 C23	^{181}WS**K**DAPSELLR^{191}	^{90}AT**K**PSALSR^{98}	42.684	105.761	107.056	34.005	TH WT

Supplementary Table 4. Distance measurements of all possible intermolecular contacts for each interprotein crosslink obtained by XL-MS.

The table presents the interpeptide crosslinks and their respective distances (Å), measured using ChimeraX, as detected by mass spectrometry analysis. On the left side of the table, the peptides from DNAJC12 (either full-length or truncated variants) are listed alongside the corresponding peptides from TH on the right (either full-length, regulatory domains (RDs), or in the presence of dopamine) where the interpeptide crosslinks was identified. The specific residues involved in the crosslink within each peptide are highlighted in bold. Considering that TH forms a tetramer, we calculated the alpha carbon (CA) distances between all DNAJC12 peptides and their corresponding peptides for each TH monomer, with the shortest distance in each instance emphasized in green. All measurements fall within the maximum established in this work (≅35 Å) for BS3, the crosslinker utilized in this study.

References

1. Jumper, J. et al. Highly accurate protein structure prediction with AlphaFold. *Nature* **596**, 583-589 (2021).
2. Faust, O. et al. HSP40 proteins use class-specific regulation to drive HSP70 functional diversity. *Nature* **587**, 489-494 (2020).
3. McWilliam, H. et al. Analysis Tool Web Services from the EMBL-EBI. *Nucleic Acids Res* **41**, W597-600 (2013).
4. Robert, X. & Gouet, P. Deciphering key features in protein structures with the new ENDscript server. *Nucleic Acids Res* **42**, W320-4 (2014).
5. Erdos, G. & Dosztanyi, Z. Analyzing Protein Disorder with IUPred2A. *Curr Protoc Bioinformatics* **70**, e99 (2020).
6. Kyte, J. & Doolittle, R.F. A simple method for displaying the hydropathic character of a protein. *J Mol Biol* **157**, 105-32 (1982).
7. Kityk, R., Kopp, J. & Mayer, M.P. Molecular Mechanism of J-Domain-Triggered ATP Hydrolysis by Hsp70 Chaperones. *Mol Cell* **69**, 227-237.e4 (2018).
8. Bueno-Carrasco, M.T. et al. Structural mechanism for tyrosine hydroxylase inhibition by dopamine and reactivation by Ser40 phosphorylation. *Nature Communications* **13**, 74 (2022).
9. Zhu, X. et al. Structural analysis of substrate binding by the molecular chaperone DnaK. *Science* **272**, 1606-14 (1996).
10. Van Durme, J. et al. Accurate prediction of DnaK-peptide binding via homology modelling and experimental data. *PLoS Comput Biol* **5**, e1000475 (2009).
11. Gutierres, M.B.B., Bonorino, C.B.C. & Rigo, M.M. ChaperISM: improved chaperone binding prediction using position-independent scoring matrices. *Bioinformatics* **36**, 735-741 (2020).
12. Fernandez-Escamilla, A.M., Rousseau, F., Schymkowitz, J. & Serrano, L. Prediction of sequence-dependent and mutational effects on the aggregation of peptides and proteins. *Nat Biotechnol* **22**, 1302-6 (2004).
13. Baumann, A. et al. Tyrosine Hydroxylase Binding to Phospholipid Membranes Prompts Its Amyloid Aggregation and Compromises Bilayer Integrity. *Scientific reports* **6**, 39488-39488 (2016).

Uncropped gels (Supplementary Figures 6a-b, 8b, 13a-b)

Supplementary Figure 6a

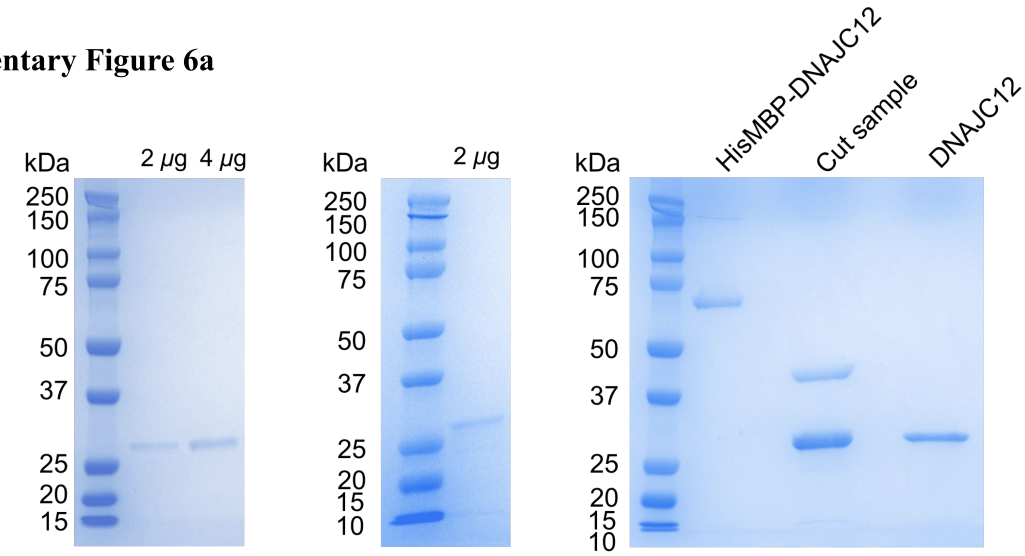

Supplementary Figure 6b

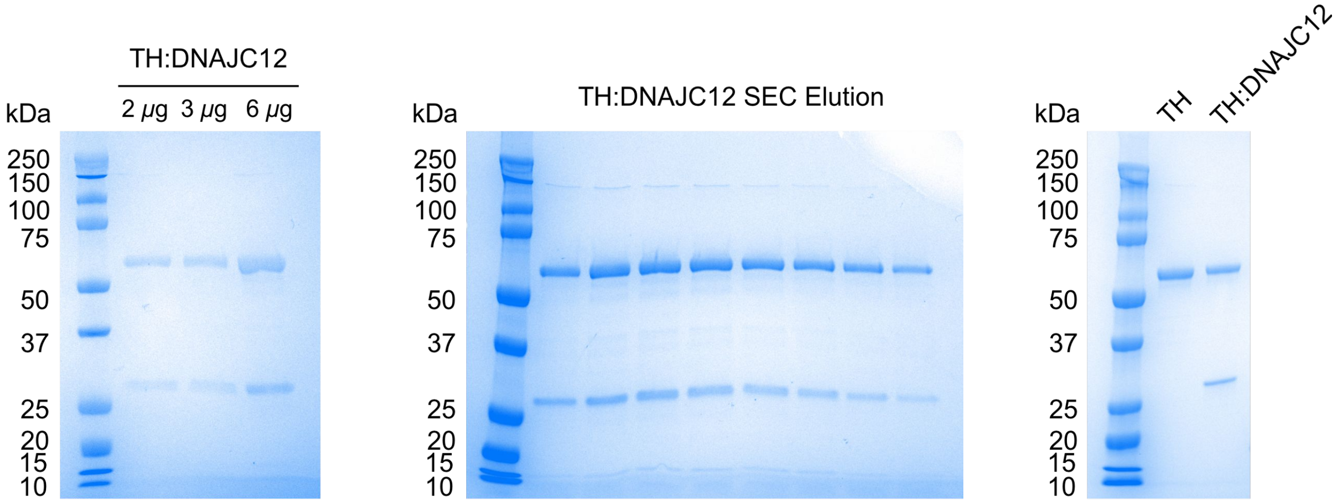

Supplementary Figure 8b

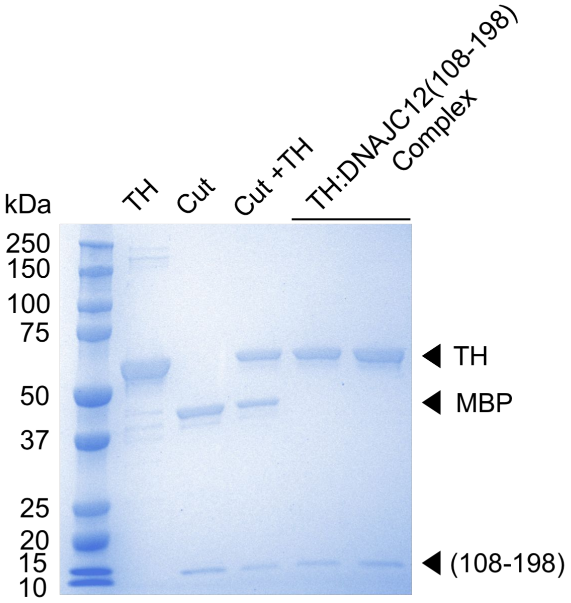

Supplementary Figure 13a

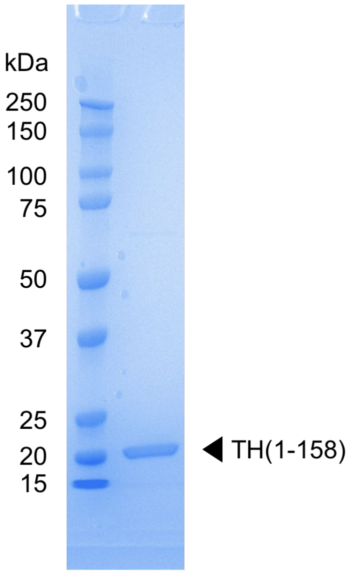

Supplementary Figure 13b

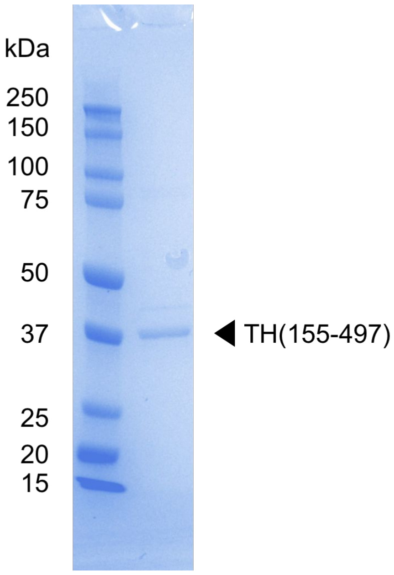

Supplement: Supplementary file 1 — Supplementary Information [file 41467_2025_57733_MOESM1_ESM.pdf]
