# Supplementary material for: Structural recognition and stabilization of tyrosine hydroxylase by the J-domain protein DNAJC12
Source: Nat Commun. 2025 Mar 20;16:2755. doi: 10.1038/s41467-025-57733-6 (PMC11926245; doi:10.1038/s41467-025-57733-6)
Supplement: Supplementary file 2 — Description of Additional Supplementary Files [file 41467_2025_57733_MOESM2_ESM.pdf]

Description of Additional Supplementary Files

File name: Supplementary Movie 1
Description: Conformational dynamics of the TH:DNAJC12 complex. This movie shows the diverse conformational states adopted by TH:DNAJC12 complex as revealed through image processing. The footage highlights the mobility of the RDs of TH and the JD and CTD of DNAJC12.

File name: Supplementary Movie 2
Description: Model of the interaction of the TH:DNAJC12 complex with Hsc70, through the DNAJC12 HPD motif. The movie shows a combined ribbon and surface model of the TH:DNAJC12 complex (PDB 6ZVP), showing the TH CDs in gray, the RDs in different shades of blue and the ODs in green. DNAJC12 is depicted with the JD in yellow and the CTD in red. DNAJC12 binds to and stabilizes TH (two DNAJC12 monomers per TH tetramer). For simplicity, only the binding of one molecule of DNAJC12 to one dimer of RDs of the tetramer is shown. The RDs contain the βstrand regions ^{82}LNLLLFSP^{88} (pink sticks) and ^{129}EYFVRLE^{135} (orange sticks), shown in both TH-RDs. These regions are predicted to bind to the Hsp70 family by the Limbo and ChaperISM software, and residues 82-88 are also predicted by TANGO to be prone to forming aggregating intermolecular cross-β interactions (main text). In the TH:DNAJC12 structural model DNAJC12 shields these regions from solvent exposure. Although the JD shows very low binding affinity for TH, CTD binding to one RD (cyan) induces the positioning of the JD in close contact with the opposite RD (light blue). Then, Hsc70 recognizes TH as a client and DNAJC12, with properly oriented HPD motif, stimulates the ATPase activity of Hsc70, leading to the ADPbound closed conformation, where the SBD is very close to the aggregation-prone regions. This model suggests the possible role of Hsp70 in the TH:DNAJC12 complex, where DNAJC12 stabilizes TH and ultimately presents it to Hsp70 for processing. For model preparation see Supp. Fig 14.
